# Acceptance of food allergic children in Japanese childcare facilities

**DOI:** 10.5415/apallergy.0000000000000147

**Published:** 2024-06-14

**Authors:** Keiko Shimazaki, Keiko Tsukasaki, Kaoru Kyota, Tomoya Itatani

**Affiliations:** 1 Division of Health Sciences, Graduate School of Medical Sciences, Kanazawa University, Kanazawa, Ishikawa, Japan; 2 Faculty of Health Sciences, Institute of Medical, Pharmaceutical and Health Sciences, Kanazawa University, Kanazawa, Ishikawa, Japan; 3 School of Nursing, Faculty of Medicine, University of Miyazaki, Miyazaki, Japan

**Keywords:** Childcare facilities, collaborating, epinephrine, food allergy, management

## Abstract

**Background:**

Food allergy management systems are important for childcare facilities to accept children with food allergies prescribed epinephrine (epinephrine-treated children). The relationship between the food allergy management system of a childcare facility and the willingness of children attending the facility to accept epinephrine is unclear. We believe that childcare facilities that cooperate with local health and medical institutions are more willing to accept children receiving epinephrine.

**Objective:**

This study aimed to clarify the actual situation of epinephrine acceptance in children attending childcare facilities across Japan and the factors associated with their willingness to accept them.

**Methods:**

Between May and June 2021, 686 childcare facilities across Japan were selected and surveyed. To analyze the factors related to the willingness to accept epinephrine children attending childcare facilities, the facilities were classified into 2 groups, “willingness to accept” and “no willingness to accept,” and their attributes and characteristics, food allergy management system, cooperation with relevant organizations and ability to cooperate were compared by univariate analysis.

**Results:**

A questionnaire-based survey of 162 centers revealed that 18.2% of the centers had accepted children receiving epinephrine and 74.7% were willing to accept them. There was a significant association between the willingness to accept epinephrine in children and satisfaction with the food allergy management system, availability of childcare consulting agencies, and ability to work with healthcare organizations, which differed.

**Conclusion:**

The results highlight the importance of strengthening the management system of facilities and cooperating with relevant institutions for epinephrine children to live safely in the community.

## 1. Introduction

Food allergies in children are life-threatening diseases worldwide, with a particularly high incidence in children under 5 years of age [1]. The prevalence of food allergies in the United States was reported to be 6.3% in 5,429 children aged 0 to 2 years and 9.2% in 5,910 children aged 3 to 5 years [2].

There have been few studies on food allergy management systems in Japanese childcare facilities. A study that included 15,722 childcare facilities reported the prevalence of food allergies in children aged <6 years to be 4.0% [3]. Over 50% of infants and toddlers in Japan attend childcare facilities, which include not only healthy children but also those with food allergies and other diseases, making them important places for children in the community to live together.

Preventing and addressing potentially life-threatening anaphylaxis are important for food allergy management. Toward this, guidelines have been developed in the United States, Canada, Europe, and Japan [4-7]. However, in a survey that included 12,181 preschools in the United States, 18.0% of the facilities had serious incidents of anaphylaxis within a span of 1 year [8]. In Japan, 7.6% of children had at least one incident of anaphylaxis within approximately 11 months in 15,722 childcare facilities [3]. When anaphylaxis is suspected, epinephrine autoinjection, which is the first-line treatment, should be administered without hesitation [6]. In most countries, epinephrine autoinjectors are approved for use by personnel and others in childcare facilities. In Western countries, it is recommended that epinephrine autoinjectors be stocked for emergency use by anyone in childcare facilities [9]. In the United States, 52% of facilities that enroll children aged 0 to 17 years have epinephrine stocks [10]. In Japan, however, epinephrine autoinjector is approved as a prescription drug for children with food allergies owing to the universal health insurance system and access to hospitals; stocking nonprescription epinephrine in facilities is not permitted. Therefore, the use of epinephrine in childcare facilities differs across countries.

Of 168 children with food allergies attending hospitals in Japan, 12% were unable to attend childcare facilities of their choice because of a history of anaphylaxis and a significant number of restricted foods [11]. Among 45,806 children with food allergy (FA) in childcare facilities, 5,123 (11.2%) were prescribed epinephrine and only 22.5% were brought to the facility [12]. Children with food allergies and a history of anaphylaxis or a prescription for epinephrine were either denied access to the facility or refused to bring them because of an inadequate food allergy management system at the facility. However, the relationship between the food allergy management system in childcare facilities and the willingness of such children to attend childcare facilities is unclear.

For follow-up visits after medical examinations, childcare facilities communicate with commissioned physicians [13, 14]. They also receive advice from public health nurses regarding children and healthcare [15]. Collaboration with healthcare providers is recommended for the management of food allergies in childcare facilities [16, 17] and is necessary when acquiring knowledge and skills regarding epinephrine autoinjectors and responding appropriately in the event of an anaphylactic outbreak. Childcare support and welfare for children with food allergies and their parents are important. We believe that daily collaboration with healthcare institutions, childcare organizations, and welfare organizations is related to the willingness of childcare facilities to accept children needing epinephrine autoinjectors.

This study aimed to clarify the actual situation of acceptance of children needing epinephrine autoinjectors attending daycare facilities across Japan and to understand the factors related to their willingness.

## 2. Methods

### 2.1. Subject of the study

**Figure 1.**
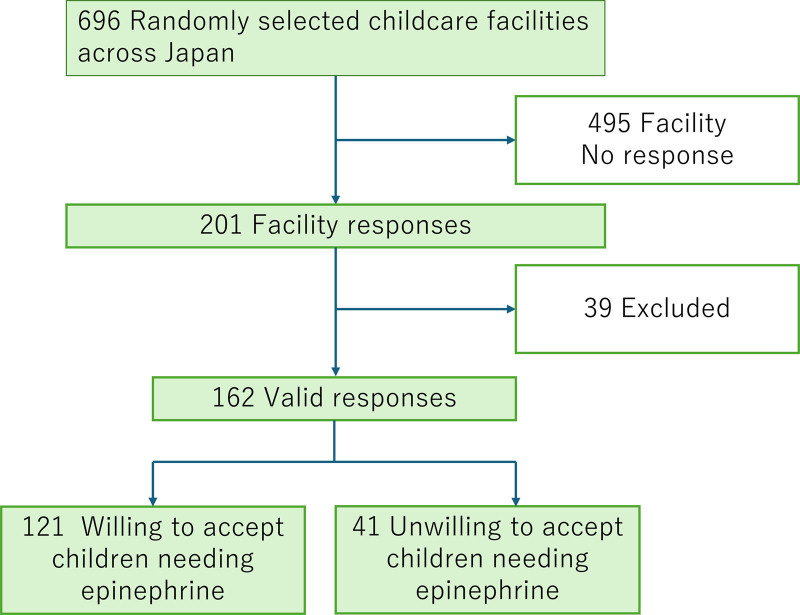
Flow chart of participants in the present study.

We randomly selected 696 regional locations from 32,954 childcare facilities in Japan. These included 418 nursery schools (64.2%), 154 certified childcare centers (23%), and 124 kindergartens (12.8%) (Fig. 1). The participants were representative of each facility. The effect size and power of the participants were checked using the G*Power 3.1 (Düsseldorf, North Rhine-Westphalia, Germany).

### 2.2. Survey contents

#### 2.2.1. Respondent attributes and facility characteristics

Gender, age, position, and years in the respondents’ offices were investigated. The type of facility, number of children, number of staff members, and the presence of resident nurses and contracted doctors were investigated.

#### 2.2.2. Food allergy status of children

The number of children with food allergies, the number of children taking internal medications and epinephrine, the status of acceptance of children with food allergies, and any experience in dealing with food allergy symptoms within the past year were surveyed.

#### 2.2.3. Willingness to accept children needing epinephrine

The willingness to accept children needing epinephrine was surveyed on a 6-point scale ranging from very willing to not at all willing. Institutions that were unwilling to accept children needing epinephrine were asked about the reason.

#### 2.2.4. Food allergy management system

We surveyed food allergy response management, emergency action plans, individual health plans for children with food allergies, existence of food allergy training in the facility, and emergency response. For emergency response, we used a confidence survey for anaphylaxis management [18] as a reference and asked respondents to indicate their confidence in 6 items: the ability to judge allergy symptoms, anaphylactic shock, injecting epinephrine at the right time, using the correct procedure, decision to call for an ambulance, and providing primary life-saving treatment.

#### 2.2.5. Collaboration with local institutions

We surveyed the state of implementation and degree of satisfaction with collaboration with healthcare organizations (such as medical institutions and health centers). The status of implementation of collaboration was judged as “fully collaborative” for facilities that answered “very collaborative” or “well collaborative” and “not collaborative” for facilities that answered “somewhat collaborative” to “not collaborative at all.” Satisfaction with collaboration was assessed on a 3-point scale: very satisfied, somewhat satisfied, and not satisfied.

The availability of childcare consultation institutions (including childcare support and child welfare) was also assessed.

#### 2.2.6. Ability to collaborate

To evaluate the facility on ability to collaborate with multiple professions, we used the Japanese version of the Interprofessional Collaboration Competency Scale for children with medical complexity [19], with the permission of the authors. This scale consists of 12 items, with 4 questions in each of the 3 domains: information sharing, resource development, and creative networking (networking). A 4-point scale (0–3) with scores for the subitems and a total score (36-point scale) was used, with higher scores indicating a greater ability to collaborate with multiple professions that have been proven to be reliable and valid.

#### 2.2.7. Survey period

The study was undertaken between May and June 2021.

### 2.3. Method of analysis

To analyze the factors related to the willingness to accept children needing epinephrine attending childcare, we classified the respondents into 2 groups: “willing to accept” (very willing to accept to somewhat willing to accept) and “not willing to accept” (somewhat unwilling to accept to not willing to accept at all) and compared their attributes and characteristics, FA management system, collaboration with related organizations, and interprofessional collaboration competency. The results were compared using a univariate analysis. SPSS ver. 23 (IBM Corp., Armonk, NY) was used for the analysis, and the significance level was set at 5%.

### 2.4. Ethical considerations

Ethical approval for this study was provided by the Kanazawa University Medical Ethics Review Committee (Approval No. 1003-1), 13, Takara-town, Kanazawa-city, Ishikawa 920-0934, Japan (Chairperson Dr. Masahiko. Tsuka) on January 25, 2021.

## 3. Results

The questionnaire was distributed to 696 facilities, of which 201 responded (response rate, 28.9%). Of these, 39 facilities that did not respond to the primary questionnaire were also excluded. Thus, 162 facilities were analyzed (valid response rate: 80.6%).

### 3.1. Facility attributes, characteristics, and collaboration status

Of the 162 facilities, 96 (59.3%) were nursery schools, 49 (30.2%) were childcare centers, 14 (8.6%) were kindergartens, and 3 (1.9%) were combined daycare center kindergartens. The total number of children was 103.4 ± 54.9 (Table 1). There were residential nurses in 79 facilities (48.8%) and contract doctors in 139 facilities (85.8%) (Table 1).

**Table 1.** Subjects and characteristics by willingness to accept children needing epinephrine

Items	Category		Willingness to accept			
		n = 162	Yes, n = 121	No, n = 41	*χ*^2^/*t*	*P*
Subjects’ characteristics						
Sex, n = 161*†	Male	25 (15.5)	19 (15.7)	6 (15.0)	0.011	0.915
	Female	136 (84.5)	102 (84.3)	34 (85.0)		
Age§		53.0 ± 10.2	52.9 ± 10.3	53.3 ± 9.9	0.217	0.828
Position, n = 161*	President	102 (63.4)	76 (62.8)	26 (65.0)	NA	
	Vice-president	13 (8.1)	9 (7.4)	4 (10.0)		
	Other	46 (28.6)	36 (29.7)	10 (25.0)		
Number of years in position‡		8.7 ± 9.2	8.7 ± 9.5	8.7 ± 8.2	0.002	0.999
Facilities’ characteristics						
Number of enrolled children	50 or less	24 (14.8)	18 (14.9)	6 (14.6)	NA	
	51–100	63 (38.9)	45 (37.2)	18 (43.9)		
	101–150	45 (27.8)	35 (28.9)	10 (24.4)		
	151 or more	30 (18.5)	23 (19.0)	7 (17.1)		
Average number of staff§		28.2 ± 12.5	29.0 ± 13.1	25.9 ± 10.4	1.533	0.129
Enrollment of resident nurse†	Yes	79 (48.8)	60 (49.6)	19 (46.3)	0.129	0.857
	No	83 (51.2)	61 (50.4)	22 (53.7)		
Enrollment of contracted doctor†	Yes	139 (85.8)	101 (83.5)	38 (92.7)	2.133	0.144
	No	23 (14.2)	20 (16.5)	3 (7.3)		

Values are n (%) or mean ± standard deviation. NA are those that were not tested due to distribution bias.

* The number excludes subjects that did not answer this question.

† *χ*^2^ test.

‡ Student *t* test.

§ Welch *t* test.

In all, 156 facilities (96.3%) collaborated with other community institutions. The main collaborating institutions included 155 (95.7%) local medical institutions and 136 (84.0%) public health centers (multiple responses).

### 3.2. Status of food allergy children, acceptance of children needing epinephrine, and food allergy management system

Of the 162 facilities with 16,745 children, 769 (4.6%) had food allergies. A total of 148 facilities (91.4%) had children with food allergies; 36 (22.2%) of these experienced allergy onset within 1 year, but none of them received epinephrine, and one requested an ambulance.

Furthermore, among the 148 facilities, 67 (45.3%) maintained oral medications for children with 769 FA children and 153 children (19.9%) brought their own medication.

Of the 148 facilities, 27 (18.2%) accepted children needing epinephrine; of the 769 food allergy children, 33 (4.3%) were children needing epinephrine.

There were 121 facilities (74.7%) willing and 41 facilities (25.3%) unwilling to accept children receiving epinephrine. The reasons for not accepting epinephrine in children at the 41 facilities were lack of knowledge (25, 61.0%), lack of a dedicated nurse (20, 48.8%), anxiety about administering epinephrine (18, 43.9%), and lack of manpower (9, 22.0%) (multiple responses).

Of the 162 facilities, 130 (80.2%) had a person responsible for the food allergy response, 138 (85.2%) had an emergency action plan, 125 (77.2%) had an individual health plan, and 116 (71.6%) had in-facility food allergy training.

### 3.3. Willingness to accept children needing epinephrine and related factors

#### 3.3.1. Food allergy management system

Compared with facilities that were willing to accept children, those who were not willing to accept children had significantly higher percentages of no emergency transport manuals (26.8%), no individual action plans (39.0%), and no in-facility food allergy training (46.3%).

All 6 items surveyed as emergency responses were associated with willingness to accept. Compared with facilities that were willing to accept patients, those who were unwilling to accept patients were 14.6% less able to determine allergy symptoms, 39.0% less able to determine anaphylactic shock, 82.1% less able to inject epinephrine at the appropriate time and procedure, 15.0% less able to determine the call for an ambulance, and 29.3% less able to perform primary life-saving procedures. These were significantly higher (Table 2).

**Table 2.** Relationship between willing to accept children needing epinephrine and food allergies management system

Items	Category		Willingness to accept			
		n = 162	Yes, n = 121	No, n = 41	*χ* ^2^	*P*
Have food allergy children†	Yes	148 (91.4)	109 (90.1)	12 (9.9)	0.985	0.521
	No	14 (8.6)	39 (95.1)	2 (4.9)		
Have food allergy managers‡	Yes	130 (80.2)	99 (81.8)	31 (75.6)	0.745	0.388
	No	32 (19.8)	22 (18.2)	10 (24.4)		
Have emergency action plans‡	Yes	138 (85.2)	108 (89.3)	30 (73.2)	6.279	0.012*
	No	24 (14.8)	13 (10.7)	11 (26.8)		
Have individual health plans‡	Yes	125 (77.2)	100 (82.6)	25 (61.0)	8.159	0.004**
	No	37 (22.8)	21 (17.4)	16 (39.0)		
Have trainings for food allergies‡	Yes	116 (71.6)	94 (77.7)	22 (53.7)	8.695	0.003**
	No	46 (28.4)	27 (22.3)	19 (46.3)		
Emergency response						
Able to determine anaphylactic symptoms‡	Yes	151 (93.2)	116 (95.9)	35 (85.4)	5.345	0.031*
	No	11 (6.8)	5 (4.1)	6 (14.6)		
Able to determine anaphylactic shock‡	Yes	134 (82.7)	109 (90.1)	25 (61.0)	18.148	<0.001***
	No	28 (17.3)	12 (9.9)	16 (39.0)		
Able to inject Epinephrine at the appropriate time, n = 157‡§	Yes	98 (62.4)	91 (77.1)	7 (17.9)	43.749	<0.001***
	No	59 (22.9)	27 (22.9)	32 (82.1)		
Able to inject Epinephrine with correct procedure, n = 157†§	Yes	98 (62.4)	91 (77.1)	7 (17.9)	43.749	<0.001***
	No	59 (22.9)	27 (22.9)	32 (82.1)		
Able to make a decision to request an ambulance, n = 161†§	Yes	153 (95.0)	119 (98.3)	34 (85.0)	11.288	0.003**
	No	8 (5.0)	2 (1.7)	6 (15.0)		
Able to do cardiopulmonary resuscitation, n = 161‡§	Yes	137 (84.8)	108 (90.0)	29 (70.7)	8.944	0.003**
	No	24 (15.2)	12 (10.0)	12 (29.3)		

Values are represented as n (%).

† Fisher direct probability test.

‡ *χ*^2^ test.

§ The number excludes subjects that did not answer this question.

* *P* < 0.05;

** *P* < 0.01;

*** *P* < 0.001.

#### 3.3.2. Collaboration with related institutions

There was no significant relationship between the willingness to accept and the implementation of collaboration with healthcare institutions. Satisfaction with collaboration was significantly lower among facilities without the willingness to accept, with 14.6% of facilities being very satisfied compared with those with willingness to accept (*χ*^2^(1) = 9.081, *P* = 0.011, *ω* = 0.0237, 1 − *β* = 1.000). Among the facilities that were not willing to accept the child, 53.7% had a childcare consultation organization, which was significantly lower than those that were willing (*χ*^2^(1) = 5.109, *P* = 0.024, *ω* = 0.178, 1 − *β* = 0.999) (Table 3).

**Table 3.** Willingness to accept children needing epinephrine in relation to collaboration with health and medical institutions and childcare consultation facilities

Items	Category		Willingness to accept			
		n = 162	Yes, n = 121	No, n = 41	*χ* ^2^	*P*
Degree of collaboration with health and medical institutions	Quite enough	127 (78.4)	94 (77.7)	33 (80.5)	0.142	0.706
	Not enough	35 (21.6)	27 (22.3)	8 (19.5)		
Satisfaction level of cooperation with health and medical institutions†	Very satisfied	49 (30.2)	43 (35.5)	6 (14.6)	9.081	0.011*
	Somewhat satisfied	96 (59.3)	69 (57.0)	27 (65.9)		
	Dissatisfied	17 (10.5)	9 (7.4)	8 (19.5)		
Childcare consultation facilities‡	Yes	110 (67.9)	88 (72.7)	22 (53.7)	5.109	0.024*
	No	52 (32.1)	33 (27.3)	19 (46.3)		

Values are represented as n (%).

† *P* value obtained from adjusted standardized residuals by residual test.

‡ Childcare consultation facilities; childcare support and child welfare agencies.

* *P* < 0.05.

#### 3.3.3. Ability to collaborate with multiple professions

Cronbach *α* for the total score of the multidisciplinary collaboration scale for this subject was 0.89, and the subitems were 0.78 for information-sharing ability, 0.82 for resource development ability, and 0.77 for networking ability.

Facilities with no willingness to accept were significantly lower than those with willingness [*t*(160) = 2.01, *P* = 0.05, confidence interval (CI) = 0.40–4.38, *d* = 0.36, 1 − *β* = 0.499] in total score and in information-sharing ability in the subitems (*t*(160) = 2.10, *P* = 0.04, *d* = 0.36, CI = 0. 06–1.83, 1 − *β* = 0.504), and networking ability was also low (*t*(160) = 2.44, *P* = 0.02, *d* = 0.43, CI = 0.22–2.10, 1 − *β* = 0.649). The resource development capacity did not differ significantly (Table 4).

**Table 4.** Differences in willingness to accept children needing epinephrine and abilities to collaborate with multiple professions

Category		Willingness to accept			
	n = 162	Yes, n = 121	No, n = 41	*t*	*P*
Ability to share information (12-point scale)	8.7 ± 2.5	9.0 ± 2.3	8.0 ± 3.0	2.10	0.037*
Resource development skills (12-point scale)	7.9 ± 2.3	8.0 ± 2.4	7.9 ± 2.2	0.25	0.804
Creative networking skills (12-point scale)	8.1 ± 2.7	8.4 ± 2.5	7.2 ± 2.9	2.44	0.016*
Total points (36-point scale)	24.8 ± 6.1	25.3 ± 6.0	23.1 ± 6.5	2.01	0.046*

Values are represented as mean ± standard deviation, student *t* test.

* *P* < 0.05.

## 4. Discussion

### 4.1. Acceptance of food allergy and children needing epinephrine

Among 162 childcare facilities in Japan, 91.4% had children with food allergies. The percentage of children with food allergies (4.6% of total children) was similar to that report [3]. In all, 18.2% of the 162 facilities had children receiving epinephrine, which was higher (14.5% of the 110 facilities) than that in a 2013 study from the central region [18]. The number of facilities accepting children needing epinephrine may have increased because of the government’s revision of the guidelines for childcare facilities [7] and the promotion of education regarding anaphylactic responses following the death of an elementary school student in Tokyo following an anaphylactic shock after a school lunch in 2012.

An earlier study reported that 25.3% of facilities were unwilling to accept epinephrine-treated children and 88% of 129 employees of childcare facilities were concerned about handling anaphylaxis [20]. The results of our study showed that 22.2% of the facilities with children with food allergies experienced an allergy within a year and 45.3% of the facilities maintained internal medications. In addition, although 85.8% of all facilities had a commissioned physician, only 48.8% had a dedicated nurse, indicating that 20% to 30% of the facilities lacked an appropriate food allergy management system. Children below 6 years are at a high risk of developing new food allergies and such children are also at risk of developing anaphylaxis with new foods. We believe that it is important to disseminate the need to assign medical personnel, emergency transport manuals, methods for developing individual action plans, and food allergy training to childcare facilities nationwide regardless of whether they have children with food allergies.

### 4.2. Willingness to accept children needing epinephrine and food allergy management system

A relationship was noted between the children’s willingness to accept epinephrine, emergency transport manuals, individual action plans, and food allergy training in the facility. Childcare staff are not accustomed to emergency response; thus, an emergency preparedness manual would be helpful [21]. Furthermore, food allergy-induced anaphylaxis presents differently in individuals and requires individualized action plans [4]. Imparting training to 155 childcare workers in Japan for 6 months, including the use of epinephrine, improved their understanding of the symptoms of anaphylaxis and the first aid system [20]. Thus, the importance of emergency transport manuals, individual action plans, and in-facility food allergy training in childcare facilities for children requiring epinephrine has been well documented. This study is the first to demonstrate that these factors are related to a facility’s willingness to accept children needing epinephrine attending the facility.

There was a relationship between willingness to accept epinephrine in children and emergency response, with a higher percentage of facilities that were unwilling to accept epinephrine in children being less confident in their ability to respond to emergencies. Approximately 80% of the staffs were not confident in the timing and procedure for administering epinephrine, and approximately 40% were not confident in judging anaphylactic shock. A total of 110 childcare workers attended a workshop on anaphylactic response, which resulted in improved self-efficacy in emergency responses [18]. A survey on the anaphylactic preparedness of 181 staff members at a childcare facility found that 71% had previously attended epinephrine training, but 69% did not have accurate anaphylactic awareness [22]. Thus, repeated training of staff on how to respond to anaphylactic shock and the use of epinephrine is important for acquiring knowledge and skills in emergency response, and confidence in emergency response may influence the acceptance of epinephrine in children.

In the present study, 48.8% of childcare facilities had a resident nurse, and no association was found between this and children’s willingness to accept epinephrine. However, 48.8% of the facilities that were unwilling to accept children cited a lack of nurses as a reason for not accepting them. In the United States, approximately 68% of the facilities have a nurse to administer epinephrine, but only half of them have a nurse present at all times [8]. Childcare facilities with nurses have a lower burden of receiving epinephrine for children [23]. Nurses can organize food allergy training and create individualized health plans for children with food allergy [24] and their presence is important for responding to emergencies and managing children’s health.

### 4.3. Willingness to accept children needing epinephrine and collaboration with relevant local institutions

This study is the first to reveal the relationship between willingness to accept children receiving epinephrine and collaboration with relevant local institutions.

No association was found with the implementation of collaboration with healthcare institutions; however, satisfaction with the collaboration was observed. In facilities with no willingness to accept, 14.6% were very satisfied and 19.5% were not satisfied. Although there are no previous studies on childcare facility collaboration, multidisciplinary team care improved blood pressure in adult patients with hypertension compared with usual care [25]. The relationship between neighboring facilities and job satisfaction is associated with self-evaluation of multidisciplinary collaboration [26]. Based on the above, we hypothesized that childcare facilities’ collaboration with local agencies affects the willingness of staff to care not only for children in the facility but also for children in the community and is related to their willingness to accept children needing epinephrine.

Willingness to accept was associated with the presence or absence of a childcare consultation agency, and facilities that were unwilling to accept were 53.7% less likely than those willing to accept to have a childcare consultation agency. Johnston et al. evaluated 32 childcare facilities with pediatric care health consultations by a community physician or nurse practitioner for 1 year [27]. Methods of consultation and support for children with special health needs, including their own health education, abuse prevention, safe sleep regimes, and food allergies, were provided to the center directors and staff of the facilities [27]. Consequently, the quality of care provided to the children improved. Multiple professional collaborations comprise individual professional and patient relationships, professional coordination, team development, and supporting systems [28]. The need for support systems to sustain the collaborative practices of interprofessional teams has been demonstrated [29]. This suggests that it is important for childcare facilities to have a local network through which they can consult on the care of children, including those with food allergies.

Facilities that were unwilling to accept children needing epinephrine had lower information-sharing and creative networking abilities in multidisciplinary collaboration than those that were willing to accept children needing epinephrine.

Information-sharing skills are the ability to share information about different perspectives with other professionals and recognize and understand new aspects of a child [19]. To manage and care for children receiving epinephrine in childcare facilities, it is important to share information with facility staff, parents, and local healthcare providers, including daily physical condition management. Additionally, growth and development assessments, balance between facility and home activities and rest, meal menus, and removal of allergy-causing ingredients are essential. It is also important for all staff to share information with parents and family doctors regarding what needs to be done in case of an emergency [7].

Creative networking skills refer to the ability to agree with other professionals regarding the purpose, planning, and clarification of support [19]. When collaborating with other professionals, insufficient communication and unclear roles may lead to accidents [30]. If care policies and plans for children with food allergies are not agreed upon by all staff in the childcare facility, inadequate care of children needing epinephrine in an emergency can be anticipated. We believe that regularly discussing policies and plans for food allergy management with daycare facility staff, parents, and relevant community organizations to reach a common understanding and strengthen networking capabilities will lead to greater confidence among daycare facility providers in responding to emergencies.

Based on the above, we believe that improving the collaborative capacity of childcare facilities and strengthening cooperation between parents, local healthcare, and childcare consultation organizations will lead to the acceptance of epinephrine in children.

### 4.4. Study limitations and challenges

The childcare facilities that responded to this survey may have been biased toward those interested in food allergy management and those willing to accept children with epinephrine. This study investigated the self-assessment of the status of cooperation with related organizations as perceived by childcare facilities; actual cooperative activities were not clear. In future, it will be necessary to clarify the reasons for inadequate food allergy management systems in childcare facilities and suggest measures to promote regional collaboration.

To conclude, the willingness of childcare facilities to accept children needing epinephrine attending their facilities was related to the development of emergency transport manuals and individual action plans, implementation of food allergy training, confidence in responding to emergencies, and collaboration between healthcare and childcare consultation organizations. These results highlight the importance of strengthening the food allergy management system in childcare facilities and collaborating with relevant local institutions for children requiring epinephrine to live safely in the community.

## Acknowledgements

We thank the daycare facilities for their cooperation with this study. This study was conducted as part of a doctoral dissertation in the Department of Health Sciences, Graduate School of Medicine and Health Sciences, Kanazawa University, Japan.

## Conflicts of interest

The authors have no financial conflicts of interest.

## Author contributions

Conception, design, and acquisition of data: Keiko Shimazaki. Analysis and interpretation of the data: Keiko Shimazaki and Tomoya Itatani. Statistical expertise: Keiko Tsukasaki and Kaoru Kyota. Critical revision of the article for important intellectual content: Keiko Tsukasaki. Final approval of the article: Keiko Shimazaki, Keiko Tsukasaki, Kaoru Kyota, and Tomoya Itatani.
